# The RADx Tech Deep Dive and Work Package 1 Process

**DOI:** 10.1109/OJEMB.2021.3070827

**Published:** 2021-04-28

**Authors:** Michael K. Dempsey, Paul Tessier, John Collins, Elias Caro

**Affiliations:** Consortia for Improving Medicine with Innovation and Technology (CIMIT), Massachusetts General Hospital and Harvard Medical School, Boston, MA 02114 USA.; Biocomx, Dana Point, CA 92629 USA.; Consortia for Improving Medicine with Innovation and Technology (CIMIT), Harvard University, Cambridge, MA 02138 USA.

**Keywords:** SBIR, RADx, translational research, funding, commercialization

## Abstract

The RADx^SM^ Tech program was a unique funding and support mechanism to accelerate the market introduction of diagnostic tests for SARS-CoV-2, the virus that causes COVID-19. In addition to providing funding, the RADx Tech program provided unprecedented levels of non- monetary support. Applications were evaluated using a deep dive process which involved a 1- to 2-week intensive collaboration between the applicant and a team of experts from RADx Tech. The result of this deep dive was a very comprehensive understanding of the potential and risks associated with the proposed work, which was far beyond what can typically be understood in a written grant application. This detail allowed the deep dive team to provide a better-informed recommendation on how to proceed. In some instances, the recommendation was made to not fund the project; in other cases, the recommendation was made to provide the applicant with more funding or support to help maximize their probability of success. After the deep dive, the project moved to a Work Package 1 (WP1) phase that focused on further de-risking. The same RADx Tech team that conducted the deep dive also worked with the applicant through the WP1 phase of the program. This allowed for joint responsibility of the work with the common goal of rapid, successful product introduction.

## I. Background

THE RADx Tech initiative aims to speed the development, validation, and commercialization of innovative point-of-care and home-based tests, as well as improve clinical laboratory tests, that can directly detect SARS-CoV-2, the virus that causes COVID-19^1^. The goals and objectives of RADx Tech were very different from those of a typical granting program.

**FIG. 1.**
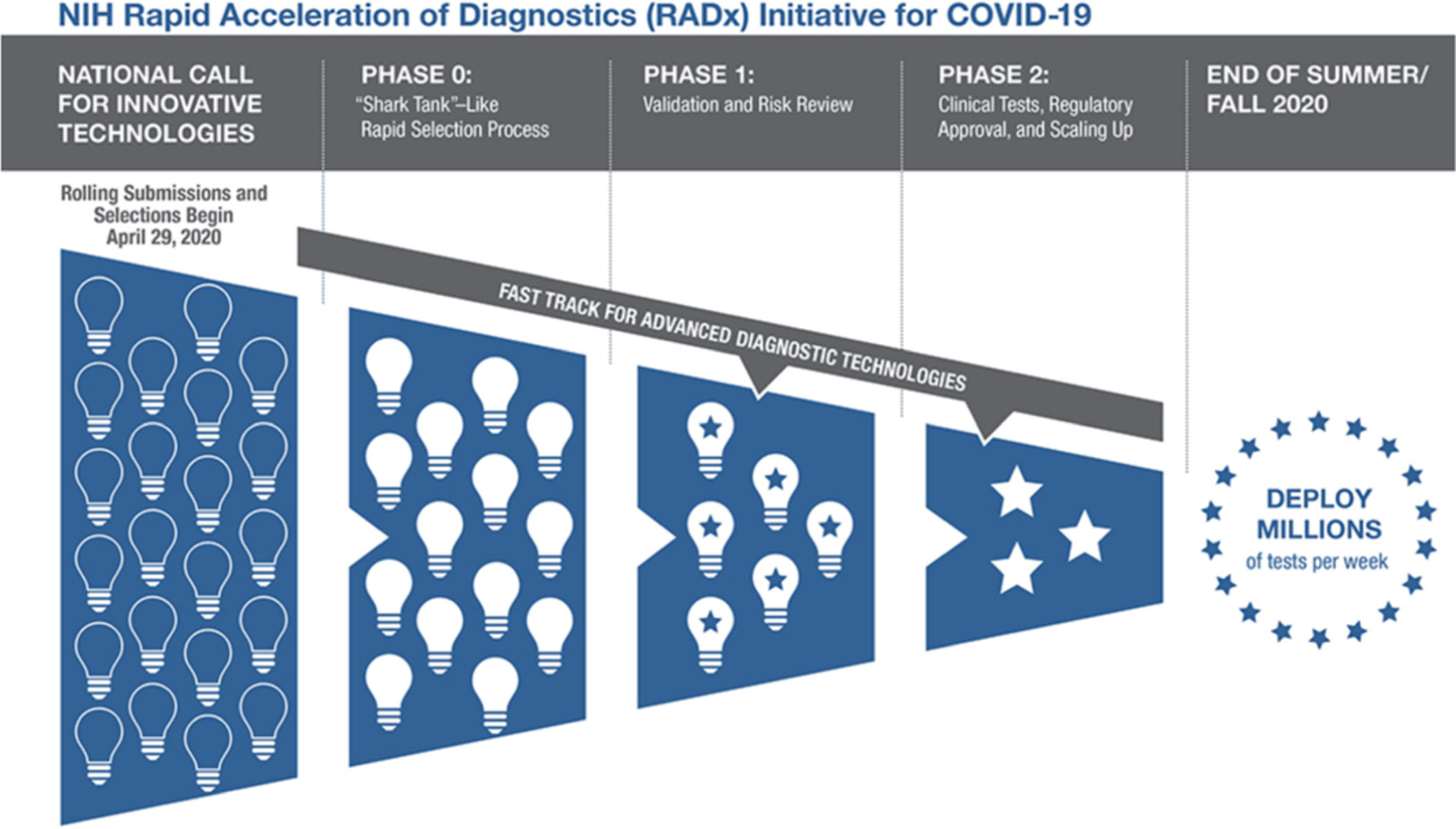
Overall RADx Tech Program^ibid.^

Whereas a typical National Institutes of Health (NIH) grant program focuses on scientific merit, RADx Tech was designed to simultaneously de-risk and accelerate the commercialization of projects that could address the national need for more and better SARS-CoV-2 testing. Consequently, the RADx Tech workflow emphasized speed and agility. Applicant teams worked closely with RADx Tech faculty who could propose changes at any time, including stopping the project. Fig. 1 is a representation of the overall RADx Tech program.

“Phase 0” is also known as the Deep Dive phase; “Phase 1” will be referred to here as the “Work Package 1” phase and “Phase 2” will be called “Work Package 2”. Applicant teams worked with RADx Tech teams to move their proposed project through Deep Dive, then Work Package 1 and then to Work Package 2 (see article by Gagliano et al. in this special issue). In this paper, we explore the details of the Deep Dive and Work Package 1 phases of RADx Tech.

## II. Team Structure

To understand these specific parts of RADx Tech, the reader must first understand the team structure that was implemented to support the overall RADx Tech program. The RADx Tech organizational structure was established to help ensure the success of the *overall RADx program*, meaning, the success of any specific applicant project was important insofar as it was part of the overall portfolio of successful projects. This allowed all RADx Tech team members to quickly and objectively shut down individual projects that were not meeting their individual objectives or the overall goals of the program. The success of the RADx Tech team was determined by the soundness of their project evaluation, their facilitation, and their project-specific recommendations, rather than exclusively by the success of the project. If a project was shut down, the RADx Tech team assigned to that project was quickly reassigned to support another project and the funding or other resources were freed up and made available to other teams. This approach, along with the relatively large number of applicants and resources available, created collaborative, supportive, and noncompetitive relationships among the RADx Tech teams as well as the shared mission of the overarching RADx Tech goal of delivering millions of tests by the end of 2020.

The project-centric organization of RADx Tech consisted of several groups (sometimes called “swat teams”), plus the project applicant.

- Team Awesome was a group of highly experienced scientists and businesspeople who managed one or more projects and had the primary responsibility of ensuring the applicant teams’ (i.e., the projects’) success; they could scale resources as necessary for any given applicant.
- A Commercialization Team vetted and provided specific resources that applicant teams required. Examples of the available resources include regulatory consultants, quality management software and support, contract research organizations, contract development organizations, and supply chain “problem solving” (see article by Gagliano et al. elsewhere in this special issue).
- A Clinical Review Team consisted of a board of clinicians and laboratorians who reviewed projects and provided early feedback on their clinical suitability (see article by Manabe et al. elsewhere in this special issue).
- VentureWell was an all-purpose team that provided contractual, personnel, financial, and other logistical support.
- The Applicant Teams were large and small companies, startups, and academic groups that applied to RADx Tech for support.

**Team Awesome** was responsible for the day-to-day operations of the projects. This team consisted of

- Six Portfolio **Executives**, responsible for a portfolio of between six and ten projects
- 32 Team **Leads** who worked intensively with the Applicant Teams under the supervision of Portfolio Executives

Nearly three-quarters of Team Awesome members had over 30 years of experience in the medtech industry and an additional 21% had between 20–30 years of experience. Over three-quarters had C-Level management experience and 13% had previously been corporate Vice Presidents. Team Awesome’s experience was typically with *in vitro* diagnostics or medical device companies. Nearly half had PhDs and 92% of them had founded their own companies. Two in three Team Awesome members had also previously invested in startup companies (note that due to conflict-of-interest concerns none could invest in, or work or consult for, RADx Tech supported companies).

This combination of deep and varied experiences allowed Team Awesome to have a unique perspective on the processes, challenges, and objectives of both large and small companies. Some Team Awesome members were retired, most were consultants, and some were starting their next company or investment fund when they joined RADx Tech. Many of the Team Leads had designed and implemented tests and testing strategies during the HIV pandemic in the 1980s; this experience proved crucial.

A key factor for all members of Team Awesome was that their financial, academic, or business success did not depend on the success of any specific RADx Tech applicant, but rather the overall success of the entire RADx Tech project. Their job was to ensure the advancement of the applicant’s project, but not to become an owner of the solution. Strict conflict-of-interest rules prohibited any member of the RADx Tech team from being an employee or consultant to any of the applicants during and for a period of time after the RADx Tech performance period. In several cases, an applicant wished to hire a member of a RADx Tech team; when this occurred, the person’s RADx Tech work immediately ceased.

The key resources that Team Awesome brought to the applicants and their projects were experience, judgement, and contacts. They could help the applicants know what to do, in what order, and who were the “best in class” resources that could help them do it. The Commercialization Team helped to bring these identified resources into the program.

To enable all the members of Team Awesome to benefit from each other and thereby the projects they facilitated, two Team Awesome meetings were organized each day:

1. An optional 30-minute open office hours where all the Team Awesome members could ask questions of other members, exchange experiences, get advice, etc.
2. A required 60-minute all-hands meeting where important processes or other issues were discussed. This all-hands meeting also frequently included speakers on topics that would help all the projects (e.g., regulatory changes or anticipated supply chain issues.)

For the first several months of RADx Tech, these meetings occurred seven days a week. Later in the program, they were reduced to 5 days a week. All project- and applicant-specific information was treated as confidential.

In addition to these structured meetings, Team Awesome members were very effective in initiating less formal communications among themselves and other groups. For example, since several RADx Tech projects used saliva as the basis for their tests, interested team members created a separate group to share information about the challenges of working with saliva. Similar groups were set up to deal with special regulatory considerations, sample acquisition, test verification challenges, and other topics of interest. Confidentiality was maintained so that, for example, information about how one project was using saliva could not be shared with a second (potentially competitive) team in the “saliva support group”. However, general information and RADx Tech support that helped all the teams was easily exchanged for the benefit of all.

In addition to regular meetings, to help ensure everyone had the most up-to-date information written information, a summary of notable COVID popular- and scientific-press articles was produced each week and distributed to the RADx Tech teams via email. The RADx Tech program also supported weekly public webinars on relevant topics; many of these were presented by members of Team Awesome.

**Portfolio Executives** (PE) were responsible for managing a group of multiple project teams. The PEs were typically the most experienced members of Team Awesome and regularly updated NIH and RADx Tech leadership about their portfolios, suggested higher-level process changes that could benefit all groups, and, most importantly, managed the Team Leads.

The **Team Leads** were assigned to a project based on how their business and technical skills could help a project. For example, if an applicant was particularly strong scientifically, but had limited business experience (e.g., a university researcher), the Team Lead would typically be someone with very strong business experience. The Team Leads led the Deep Dive Review, then continued to support the project through Work Package 1 and Work Package 2. The Team Lead and Portfolio Executive coordinated all the resources for every project and provided intimate direction and support to the funded applicants.

While not technically members of Team Awesome, a key element of success was support provided by Project Facilitators and Assistant Project Facilitators.

The **Project Facilitators** were skilled project managers with more than 20 years of experience in diagnostics or the medical industry coordinating the activities of multifunctional teams toward the goal of on-time quality product launch. Project Facilitators who had diagnostics expertise were assigned to Team Leads that came from other medical industries.

**The Assistant Project Facilitators** were selected from a national pool of Baccalaureate or Masters students in biomedical engineering with limited industry experience. This allowed them to receive valuable experience with the full product development and commercialization cycles, an experience that will positively impact their future careers (see article by DiMeo et al. in this special issue). They helped coordinate meetings, produce presentations and other project artifacts, took minutes, etc.

In the first two months, as hundreds of applications were received and triaged, and the various RADx Tech teams were recruited and formed, there was routine and ongoing training for all members of Team Awesome. This included support in the use of the project/program management tools such as GAITS and CoLab, and all the processes around RADx (see article by Collins et al. in this special issue). There was also a secure portal where all this training was maintained in video and written formats, with a coordinator specifically assigned to help new Team Awesome members quickly come up to speed. Members of Team Awesome also produced curated documents with suggestions and best practices that were a valuable resource for new members. Due to this intensive training, a new member of Team Awesome typically could be on-line in less than 24 hours.

During the Deep Dive, which ideally lasted one calendar week but sometimes took a bit longer, any given applicant received 60–100 hours of combined Portfolio Executive, Team Lead, Project Facilitator and Project Facilitator support. During WP1, additional commercialization resources were added to the team to support regulatory, quality systems and scaleup manufacturing expertise, as described elsewhere in this special issue.

Of note, RADx Tech was fortunate in that recruiting team members benefited from a few factors. First, the economic downturn during the pandemic meant that skilled people were generally available. Second, many of the more senior members (e.g., Team Awesome) were already retired or independent consultants, so they had time available and the specialized skills needed to accomplish the RADx Tech mission. Even so, essentially no one who was asked to participate in Team Awesome refused and most people said something to the effect of “I have been training my whole life to work on this problem and I will never have another opportunity to work on a Manhattan Project-like effort such as this again, so count me in for anything you need.”

## III. Radx Tech Use of the Guidance and Impact Tracking Tool (GAITS)

The Consortia for Improving Medicine with Innovation & Technology (CIMIT)^2^ is the Coordinating Center for the NIH-funded Point of Care Technology Research Network (POCTRN)^3^. The RADx Tech teams used several tools previously developed by CIMIT. One of these that proved very valuable was the Guidance and Impact Tracking Tool (GAITS)^4^. The GAITS platform was used to provide a mechanism for the teams to report the status of a project and proposed work plans in a consistent way. GAITS measures the maturity of a project along ten maturity levels (based on Technology Readiness Levels – TRLs) across four key domains (clinical/workflow, Market/Business, Regulatory, and Technology). See article by Collins et al. in this special issue for further details.

**FIG. 2.**
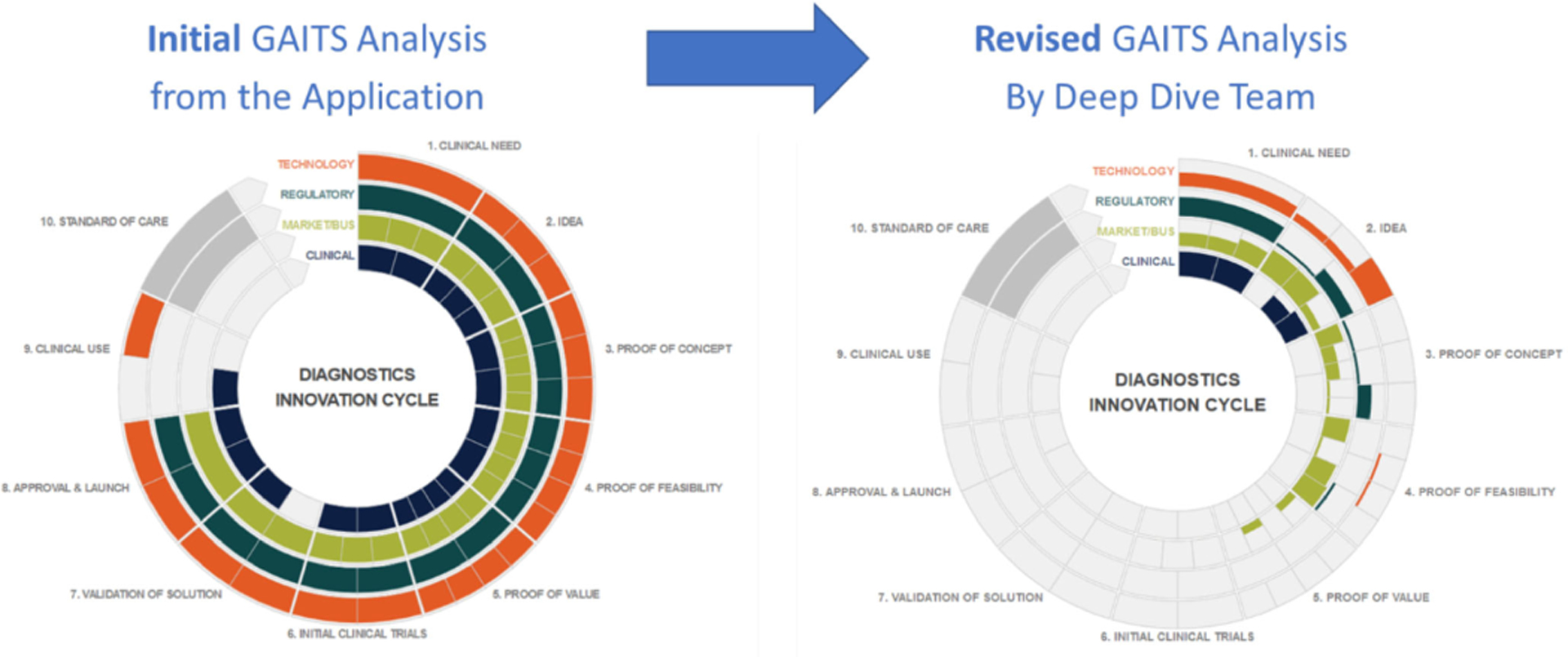
GAITS Analysis by Applicant and as Revised after Deep Dive. The standardized analysis framework and reporting format of GAITS enabled the RADx Tech team to quickly determine that this project, despite the applicant’s optimistic self-analysis, was too early for RADx Tech.

The RADx Tech team started with the applicant’s own self-assessment of their project, then after the Deep Dive the RADx Tech team compared their assessment of the project’s maturity to the original self-assessment. Often, they would find that teams, especially those with little commercialization experience, had significantly overestimated their progress and were not in a position to meet RADx Tech’s time-related goals. For example, Fig. 2 below shows one team’s incoming self-assessment on the left and the revised assessment of the Deep Dive team on the right. This project did not proceed forward.

**FIG. 3.**
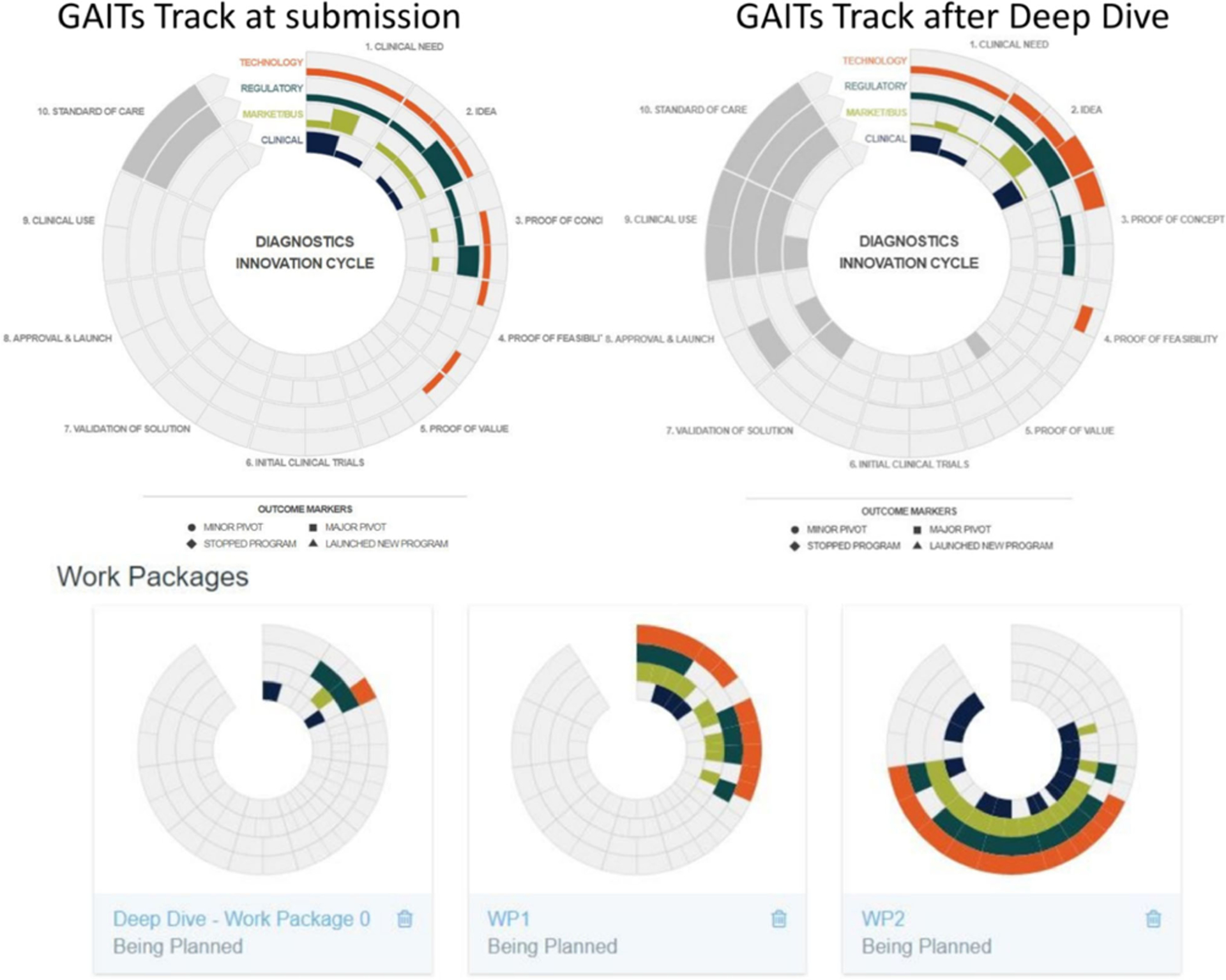
Capture of Proposed Work. GAITS allowed the teams to present in a consistent way how their WP1 and WP2 plans would advance the project to key inflection points and ultimately shipping high-performing tests. This consistent framework made it easier for the reviewers and NIH to understand and compare the various projects and how the projects were using the funds to reach uniform milestones.

For projects that continued to show promise after the Deep Dive status assessment, the RADx Tech team would work with the applicant to develop the fastest commercialization plan they could. It was very important for the RADx Tech teams to be involved in the planning so that they could bring in additional resources as needed. The workplans were captured in GAITS as a collection of deliverables, which could include custom deliverables per team. Fig. 3 shows one such example with the initial status and planned Work Packages 1 and 2.

## IV. Deep Dive

The objective of the RADx Deep Dive process was to evaluate applications in detail to determine if they met the RADx Tech program objectives. For the sake of this discussion, we will refer to the collection of all the RADx Tech resources that are working on a specific project as the “Deep Dive Team”. Unlike traditional grant application reviews, which are entirely based on the written materials submitted by the applicant, the Deep Dive Team worked very closely with the applicant team to conduct this evaluation. When an application was received, it was evaluated by the Viability Panel for a high-level fit with the program (see article by Tessier et al. in this special issue). If the Viability Panel determined there was a programmatic fit (which was the case for about 20% of the applications), the Team Lead and other members of Team Awesome were assigned to the project. This typically happened the same day the Viability Panel reviewed the project. The Team Lead then immediately contacted the applicant and began the Deep Dive process. The initial contact was typically with the Principal Investigator (PI) but given the breadth of data and support provided and required, normally the interactions with the applicant expanded to include the applicant’s CEO, Sales and Marketing Team, Regulatory Team, Manufacturing Team as well as the scientific team. If it became clear that the applicant was lacking in one of these areas (for example, many small companies did not have regulatory support), the Team Lead would work with the RADx Commercialization Team to provide the applicant with these resources.

The applicant was awarded a flat $25000 award from NIH for the Deep Dive process, which was targeted to take 1 week (although it often took longer). The award was primarily intended to support the applicant’s time in answering the Deep Dive team’s questions. This was quite an intensive effort for the Deep Dive team, which could easily spend 100 hours during the week of the Deep Dive. On average, the total compensation for the entire RADx Tech team during the Deep Dive was about $15000 per project. It was the opinion of RADx Tech leadership that this $15000 was a worthwhile investment for two reasons. First, the Deep Dive helped to ensure that we had a realistic view of the technology and the applicant. Second, it identified exactly what the project was going to need during the performance period to be successful.

## V. Deep Dive Compared To Typical Grant Process

The RADx Tech Deep Dive differed from a typical NIH or other public agency grant review in several ways. First, the RADx Tech team worked closely with the applicant to verify and pressure test what was written in the application. In some cases, this included video tours of the facility (site visits were precluded by the pandemic), having detailed design and program reviews with the applicants, and having RADx Tech cores or third parties reproduce scientific results (see article by Lam in this special issue). The RADx Tech team also considered many success factors beyond the science, for example, the ability of the applicant to produce the product at the needed volumes in the required time frame. Perhaps most importantly, since the RADx Tech Deep Dive team knew they would ultimately be driving the project to success if it advanced beyond the Deep Dive stage, they could change or augment the resources requested. The Deep Dive team could significantly increase the budget, add extra deliverables and milestones, and change the timeline. Of course, all of this occurred working closely with the applicant and NIH; the latter was ultimately responsible for determining and approving the final funding amount, based on the recommendations of the Deep Dive team and affirmed by the Steering Panel (see article by Tessier in this special issue).

In some ways, the Deep Dive process was more like the diligence a venture capitalist (VC) might do before investing in a company. This intensive but collaborative interaction has a goal of attempting to understand the risks of the project and hence maximize the probability of success. However, unlike most VC due diligence, since the RADx team doing the Deep Dive knew they would ultimately be responsible to help execute the project success (not just determine the needed level of funding), they tended to think more like business operators or founders rather than funders. This meant the diligence was very hands-on and detailed and frequently included the RADx Tech team helping to improve or refine the application and workplans.

It should be noted that there was a huge diversity in applicants from multi-billion-dollar publicly traded companies, to 2-person startups, to academic labs. These applicants all had their own business planning approaches; from the ISO-13485 compliant systems of the big companies to the “pitch decks” of the small companies. Each of these business planning approaches had their strengths and weaknesses; hence, the required diligence from the Deep Dive teams was different. Due to the experience of Team Awesome in both big and small companies, they tended to know the strengths and weakness of these different business planning approaches and therefore what risks to dig into. In general, the big companies tended to have very detailed plans that did not take enough risk or were not fast enough; they needed to be encouraged to think more like a startup. The Deep Dive team needed to figure out and justify why a big, well-funded company deserved NIH support. For example, if a larger company had a high-performing test but couldn’t produce enough of them because their production lines were fully committed to other products, the Deep Dive team may suggest funding a new production line at that company to increase capacity. The smaller companies frequently missed important business issues (e.g., how will we sell this?) and so they needed help in different areas. In all cases, the Deep Dive team tried to understand and bring to bear RADx Tech resources to compensate for these weaknesses.

## VI. Evaluation Criteria for Deep Dive

Deep Dive teams assessed projects using evaluation criteria outlined in the RADx Tech solicitation^5^. Projects were assessed to determine the potential of the proposed solution to meet each of the following criteria by late summer, 2020 or soon thereafter:

1. **Technical:** Can the technology be developed to the highest levels of analytical performance (e.g., sensitivity, specificity, dynamic range, limit of detection, reliability, accuracy, speed and throughput) as well as operational performance, such as patient- and user-friendly design, alternative sampling strategies (saliva, exhaled breath, etc.), optimization of swab materials and test reagents, mobile-device integration, increased accessibility and home-based use? Do these technical/design advances reduce barriers to expanding national testing capacity and provide clear advantages over current approaches?
2. **Clinical:** Does the proposal provide a realistic approach to increasing SARS-CoV-2 testing in a way that can be rapidly integrated into and adopted by the healthcare system?
3. **Commercial:** Assuming the technology works as anticipated, can it be implemented and made available/manufactured at scale in an economically viable way?
4. **Regulatory**: Are there feasible plans to perform the studies required to obtain FDA Emergency Use Authorization (EUA) and to subsequently obtain FDA clearance?

In addition, to be considered, the proposed technology must directly detect SARS-CoV-2. Both early stage and advanced stage projects were eligible.

The Deep Dive team used a standard presentation outline to present their assessment of an applicant and their project against these criteria to the Steering Panel in the form of a 30-to 45-minute presentation from the Team Leader based on the standard template. The applicant was not present for this presentation, which allowed the Steering Panel and the Deep Dive teams to have very candid conversations. Interestingly, most of the discussions tended to be around how to help overcome challenges that the Team Lead highlighted: Can we provide more funding? Does the RADx Tech Commercialization Team need to provide additional tools to the applicant? Can we encourage a partnership of the applicant with another organization? After the Team Lead’s presentation, each voting member of the Steering Panel provided their recommendation using a numerical scale for each of the above criteria and in a brief written summary.

## VII. Work Package 1

As noted above, if the Deep Dive Team concluded that the project met the evaluation criteria, and the Steering Panel generally recommended to NIH that the project advance to Work Package 1. For very advanced projects, the Steering Panel could suggest skipping Work Package 1 and jumping directly to Work Package 2; this occurred 9% of the time. The Steering Panel could also suggest the project be rejected or “redirected” to a different source of funding; this occurred with 56% of the projects undergoing Deep Dives. The NIH Funding Panel then made the ultimate choice among these options.

As soon as the NIH recommended that a project advance to Work Package 1, the RADx Tech team and the applicants began an approximately one-month phase of work. The Deep Dive Team also recommended a budget for Work Package 1, which given the time-bound nature of the work, tended to be in the $1 – $5M range. Assuming NIH approved this budget, the project was assigned to one of the POCTRN Centers^3^. The Center worked with the applicant to complete the Just in Time paperwork required to issue the sub-award and then subsequently issued the award to the applicant for the requested amount as a grant. Like all NIH grants, the award reimbursed for completed work. Therefore, if the project was not meeting milestones, or if the project could not be appropriately de-risked, the grant would be terminated, and no additional monies spent. The total hours of a RADx Tech team during this (typically) month-long Work Package 1 phase averaged about 50 hours a week, or 200 hours total. The expense of the effort of the RADx teams was budgeted independently of the grant amount the applicant received.

The output of the Deep Dive was generally a broad review of the overall project with some specific deeper analysis in key risk areas. The same team that did the Deep Dive worked with the applicant during Work Package 1, thus allowing for a smoother and more efficient transition than would be required if a new facilitation team were assigned. The team then continued their work with the applicant during Work Package 1 to reduce the risks to the project and establish a feasible plan for bringing the project to the market.

Unlike a typical grant, where the granting organization primarily provides funding and the grant recipient does all the work, in the RADx Tech program, the RADx Tech team accomplished a great deal of work during WP1. They decided which additional resources needed to be brought to bear on the problem and they worked with the team to think through scientific, business or other strategic advice. The RADx Tech teams were very much “in the trenches and at the bench” with the applicant during Work Package 1; they worked to troubleshoot and solve problems as they arose. They advocated for the project when there were technical difficulties or schedule slips. Each week, the Portfolio Executive and Team Leads presented the progress of their projects to RADx Tech and NIH leadership, as well as their peers. Many times, this led to insightful but difficult questions (Why is this taking so long? Are you sure the technical problem is really solved? Will this really be a competitive product? etc.)

The fact that the RADx Tech teams became embedded in the project teams with the applicant and did a significant amount of work also differentiates RADx Tech from a typical startup incubator or accelerator. These incubators provide space, advice, and sometimes financial support to their clients, but they tend to treat their clients more as investments than their “own” company. The leadership of the incubator typically does not assume an operational role in the companies within their organization. While some incubators are thematically driven, it is rare for them to have deep and specific domain expertise to support the development of their client companies.

During Work Package 1, the RADx Tech team worked to assess the project against an advanced set of evaluation criteria and to help the applicant best meet those criteria. To exit Work Package 1 and move on to Work Package 2, projects were assessed in the follow five areas:

1. Basic Performance
  - Specificity, sensitivity, LOD testing performed as agreed with the Verification Core (see article by Lam et al. in this special issue).
  - Results should provide high confidence the test meets or exceeds the performance of comparable existing diagnostic tests and meet most recent EUA requirements (matching CLIA classification).
  - EUA approval information, if available.
2. Workplan for Commercialization
  - Scaleup plan for production volumes including forecasts, timeline, milestones, supply chain needs, and other ramp-up considerations
  - Key commercial partners, contractors, vendors, and service providers secured including quotes/estimates and support/commitment letters as appropriate
  - High-level clinical studies plan with Clinical Studies Core (see article by McManus et al. in this special issue)
  - Schedule and budget through market entrance including pilot production, verification, validation, scaleup, and distribution
  - The workplan needed to be reviewed by an ad hoc panel selected from a subset of the members of Team Awesome who had experience related to the work at hand.
3. Embodiment of the proposed product.
  - Product should have testing evaluation on process model if production model not available
4. Quality Management System (QMS) requirements.
  - Established Quality Management System appropriately reflecting current state of development with plans to establish complete QMS if not currently completed
5. Use Case Analysis
  - Preliminary CLIA classification by FDA and/or RADx Tech regulatory experts. (High Complexity, Moderate Complexity, Waived, or Home/OTC)
  - Competitive Analysis versus other EUA products, if applicable, showing differentiation and benefits of the product such as improved performance, reduced cost, better use model, and/or increased production compared to currently available tests
  - Adoption and economic viability analysis for proposed use cases (including capital cost, staffing, test cost, false positives, false negatives)
  - Clinical Assessment for use case fit with Clinical Review Panel, as appropriate
  - External “End User” assessment evaluating whether the proposed test, including CLIA classification, will be feasible to deploy and meet needs, including a market analysis comparing potential production volume to potential market demand

## VIII. Challenges During Deep Dive and Work Package

While the Deep Dive and Work Package 1 processes had many advantages, they also had some challenges. Generally, these fell into one of two categories: uncooperative project teams and administrative delays.

By absolute numbers, the “problematic teams” were small: approximately 4 of the 138 projects. Universally the challenges encountered were related to dysfunctional applicant organizations. For example, in one case there was a very “siloed” applicant organization where the various departments did not trust or communicate with each other, so information was difficult to come by, slow to obtain, and sometimes contradictory. This was very frustrating to the RADx Tech team. In another circumstance, the company misrepresented data to RADx Tech on several occasions; when this was discovered, the project was terminated. In this second circumstance, one can imagine how this may have gone on for months or years with a more typical granting mechanism; with RADx Tech this was discovered relatively quickly. The identification of these dysfunctional teams was beneficial in that they were terminated from the program relatively quickly.

A bigger issue was administrative delays. As noted above, speed was a crucial element of the RADx Tech program. Government agencies (NIH, CDC, FDA, etc.) and academic Research Management offices were asked to make decisions, approve projects, and release funds at unprecedented speeds compared to their traditional decision-making timelines. However, these decisions were still sometimes painfully slow by RADx Tech timeline expectations. For example, the RADx Tech objective for a Work Package 1 timeline was 4 weeks; in some circumstances it would take NIH 2 or more weeks just to approve funding, then the academic center who was issuing the subaward another month to release the funds. While both of these periods of time would be seen as quick by traditional grant-funding practices, the reader will note that in this case the 4-week project was 6 weeks behind before it started. Holidays were also frequently a sore point; all the RADx Tech teams and most of the NIH team worked 7 days a week for the first 6 months, including holidays and weekends. The RADx Tech team also expected the applicant team to do the same, and virtually all of them did. Yet it was frustrating when decisions could not be made or funds could not be released because a key person at a Research Management office went on vacation and decisions could not be made in their absence.

Some applicants were able to compensate for this and keep on schedule by self-funding the work at their risk. Other applicants, typically startup companies, did not have the internal resources to do this, so their project timeline slipped.

If a RADx Tech-like structure is considered in the future, the ability to overcome these disconnects between “government/academic speed” and “startup speed” should be solved from the beginning.

## IX. Conclusion

The RADx Tech Deep Dive and Work Package 1 teams and procedures were unique in that they brought unprecedented resources to help any given project and the overall program be successful. The RADx Tech teams engaged deeply with the applicants; they were not simply reviewers, gate keeper or advisors but key drivers for the success of the projects. They also engaged broadly with project teams, providing support for science, business, manufacturing, management, regulatory and other challenges. The RADx Tech teams’ shared objective was to ensure the success of the applicant projects, but at the same time not becoming owners of the solution. If a given project did not meet the criteria of the RADx Tech program, the teams moved on to other projects; the RADx Tech team members personal success was therefore not tied to the success of any given project.

The unprecedented need created by the COVID-19 pandemic, along with the unique tools, organizational and team structures put in place, allowed the RADx Tech program to enable millions of new tests to be brought to the market at unprecedented speed. We plan to report the quantitative value of RADx Tech in general, and the Deep Dive and Work Packaged 1 processes specifically, in future papers. The results of these studies are anticipated to support the adaptation of RADx Tech to other areas of translational research.

## Impact Statement—

The Deep Dive and Work Package 1 phases of RADx Tech enabled comprehensive project reviews and project de-risking at unprecedent speeds (typically 1 to 2 weeks to review and 1 month to de-risk).
